# Kernel based methods for accelerated failure time model with ultra-high dimensional data

**DOI:** 10.1186/1471-2105-11-606

**Published:** 2010-12-21

**Authors:** Zhenqiu Liu, Dechang Chen, Ming Tan, Feng Jiang, Ronald B Gartenhaus

**Affiliations:** 1 University of Maryland Greenebaum Cancer Center, 22 South Greene Street, Baltimore, MD 21201, USA; 2 Department of Preventive Medicine and Biometrics, Uniformed Services University of the Health Sciences, Bethesda, MD 20814, USA; 3 Department of Pathology, The University of Maryland School of Medicine, Baltimore, MD 21201, USA

## Abstract

**Background:**

Most genomic data have ultra-high dimensions with more than 10,000 genes (probes). Regularization methods with *L*_1 _and *L_p _*penalty have been extensively studied in survival analysis with high-dimensional genomic data. However, when the sample size *n *≪ *m *(the number of genes), directly identifying a small subset of genes from ultra-high (*m *> 10, 000) dimensional data is time-consuming and not computationally efficient. In current microarray analysis, what people really do is select a couple of thousands (or hundreds) of genes using univariate analysis or statistical tests, and then apply the LASSO-type penalty to further reduce the number of disease associated genes. This two-step procedure may introduce bias and inaccuracy and lead us to miss biologically important genes.

**Results:**

The accelerated failure time (AFT) model is a linear regression model and a useful alternative to the Cox model for survival analysis. In this paper, we propose a nonlinear kernel based AFT model and an efficient variable selection method with adaptive kernel ridge regression. Our proposed variable selection method is based on the kernel matrix and dual problem with a much smaller *n *× *n *matrix. It is very efficient when the number of unknown variables (genes) is much larger than the number of samples. Moreover, the primal variables are explicitly updated and the sparsity in the solution is exploited.

**Conclusions:**

Our proposed methods can simultaneously identify survival associated prognostic factors and predict survival outcomes with ultra-high dimensional genomic data. We have demonstrated the performance of our methods with both simulation and real data. The proposed method performs superbly with limited computational studies.

## Background

Survival prediction and prognostic factor identification play a very important role in medical research. Survival data normally include the censoring variable that indicates whether some outcome under observation (like death or recurrence of a disease) has occurred within some specific follow-up time. The modeling procedures must take into account such censoring. It is even more difficult to develop a proper statistical learning method for survival prediction.

Several models for survival predictions have been proposed in statistical literature. The most popular one is the Cox proportional hazards model [1-3], in which model parameters are estimated with partial log likelihood maximization. Another one is the accelerate failure time (AFT) model [4-6]. AFT is linear regression model in which the response variable is the logarithm or a known monotone transformation of a failure (death) time. There are mainly two approaches in literature for fitting a AFT model. One is the the Buckley-James estimator which adjusts censored observations using the Kaplan Meier estimator [7,8], and the other is a semiparametric estimation of AFT model with an unspecific error distribution [9-11]. However, the semi-parametric Bayesian approach based on complex MCMC procedures is computationally intensive and tends to have inaccurate results, and the Stute's weighted least squares (LS) estimator only implicitly accounts for the censored time. The model has not been widely used in practice due to the difficulties in computing the model parameters [12], and there is no nonlinear AFT model in the literature.

Kernel based methods such as support vector machines (SVM) have been extensively studied recently in the framework of classification and regression [13] in the area of pattern recognition and statistical learning. The concept of kernel formulated as an inner product in the feature space allows us to build nonlinear extensions of many linear models [14]. It would have been a potential alternative if it were not for the complexity of censoring. Moreover, LASSO type penalty and its generalized versions have been proposed for gene (variable) selection with high dimensional genomic profiles with censored survival outcomes [15-18]. However, since the sample size *n *≪ *m *(the number of variables), methods based the primary formulation with a huge m (*m *> 40, 000) are not efficient. Consequently, in current microarray analysis, what people really do is select a couple of thousands (or hundreds) of genes using filter-based methods (such as T-test) and then apply the LASSO-type penalty to further reduce the number of disease associated genes. This two-step procedure will lead to missing biologically important genes and introducing bias. The dual solution with kernel proposed in this article attempts to resolve these inadequacies by solving a much smaller *n *× *n *matrix.

In this paper, we propose a nonlinear kernel ridge regression for censored survival outcome prediction under the framework of AFT model. We also develop an efficient dual solution with adaptive kernel ridge regression for ultra-high dimensional genomic data analysis.

Unlike the weighted least square method, our model explicitly accounts for censoring. The proposed models are evaluated with simulation and real data and the prediction error of the test data.

## Results and Discussion

### Simulation Data

Simulation studies are conducted to evaluate the performance of the proposed methods under different assumptions. The following describes a method to generate input data with censored survival outcomes that emulates the mechanisms presented by the actual data.

1. Sample 12 -dimensional input data **x **with 100 training and test samples respectively from a multivariate normal distribution with mean zero and variance-covariance matrix Σ. The pairwise correlation between the *i*th and the *j*th input variables in Σ is *r*^|*i*-*j*| ^and different correlations (*r *= 0.2, 0.4, 0.6, and 0.8) will be chosen to assess the performance of the proposed method.

2. Choose the model parameters **w **= [1, 1, 1, 1, 1, 1, -1, -1, -1, -1, -1, -1]*^T^*, and generate the event time from log *T *= **w***^t ^***x***^k ^*+ *ε*, where *ε *~ *N*(0, *σ*^2^) and *σ *is determined by the signal to noise ratio (SNR = *μ_surv_/σ*). For instance, with the mean log survival time of 3, and *SNR *= 3 : 1, we have *σ *= 1. *SNR *= 3 : 1 is used in all of our simulations. Finally, *k *indicates the *k*th power of input variables, so the log survival time is associated with the input variables nonlinearly.

3. The censoring variables are generated as uniformly distributed and independent of the events. Letting *d_i _*= (*rand *+ *C*)*T_i_*, the censoring status will be *δ_i _*= *T_i _*<*d_i_*. Different Cs give a different portion of censored data. Roughly 40% - 60% censored data are produced in our simulations.

**Figure 1:**
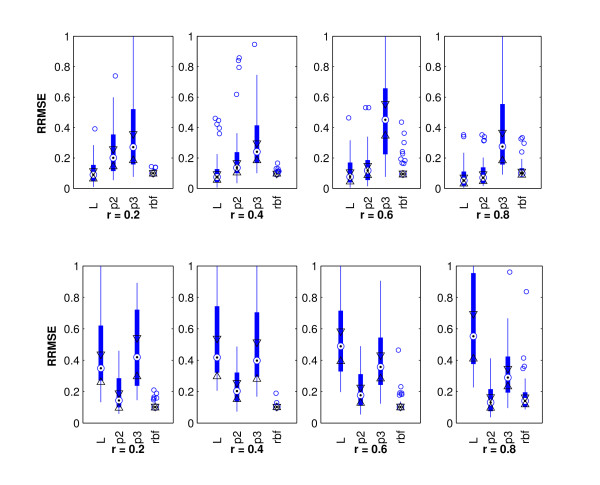
**Test RRMSE with Different Correlation Structures**. Test Relative Root Mean Squared Error (RRMSE) with Different Models and Different Correlation Structures: L - linear; p2 - second order polynomial kernel; p3 - third order polynomial kernel; and rbf - radial basis function kernel. The upper panels show the performance with the linear model (k = 1) and the lower panels show the performance with quadratic model(k = 2).

We analyze the simulation data with the proposed DKRR algorithm and build the model with training data, evaluate the performance of the model with the test data. The performance of the DKRR algorithm with different kernels and different correlation structures are shown in Figure 1. As shown in the upper panels of Figure 1, when the the survival data are simulated with *k *= 1 and the true model is linear, the linear model has the best performance with the the average relative root mean squared error (RRMSE) around 0.1. Models with the radial basis function (rbf) kernel have the second best performance with different correlation structures (r = 0.2 -0.8). Models with the third order polynomial have the worst performance with the mean RRMSE around 0.4. On the other hand, when the survival data are generated with a quadratic model with *k *= 2 as shown in the lower panels of Figure 1, Model with second order polynomial kernel and rbf kernel are the two top performers with the average test RRMSE around 0.2, and the linear model performs the worst with the largest average test RRMSE around 0.6. These results indicate that model specification is very important. A misspecified model may lead to inaccurate predictions. Finally, there are no statistical significant differences for input variables with different correlations (r = 02 -0.8).

**Figure 2:**
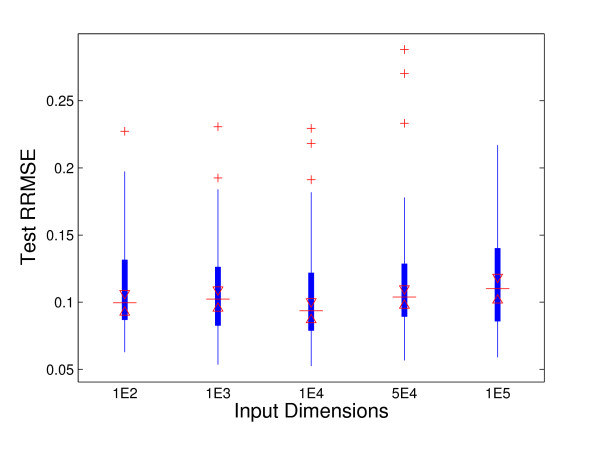
**RRMSE with Different Input Dimensions**. Test Relative Root Mean Squared Error (RRMSE) with Different Input Dimensions. The input dimensions vary from 100 to 100,000.

To evaluate the performance of AKRR method, the survival data are generated from linear model with *r *= 0.4, and different **w**s. The generated input data have the dimensions of 100, 1000, 10000, 50000, and 100000, but only 12 variables at the positions of 1, 11, 21, 31, ..., 101, 111 are nonzero with the values of [*w*_1_, *w*_11_, *w*_21_, ..., *w*_101_, *w*_111_]*^T ^*= [1, 1, 1, 1, 1, 1, -1, -1, -1, -1, -1, -1]*^T ^*, [0.2, 0.2, 0.2, 0.2, 0.2, 0.2, -0.2, -0.2, -0.2, -0.2, -0.2, -0.2]*^T^*, or [0.1, 0.1, 0.1, 0.1, 0.1, 0.1, -0.1, -0.1, -0.1, -0.1, -0.1, -0.1]*^T ^*respectively. The rest coefficients are set to 0. The random noise and rest of the variables are generated from the distribution of *N*(0, *σ*^2^), and *σ *is determined by the mean survival time and the signal to noise ratio (SNR = 3:1). The test RRMSEs with different input dimensions are shown in Figure 2. Figure 2 shows that the test RRMSEs have not changed significantly when the input dimension increases from 100 to 100000, which indicates that AKRR method performs well even with a huge number of variables. The frequencies of first 12 component variables being selected out of 100 random simulations with different **w **are given in Table 1. Table 1 shows that AKRR can correctly identify the survival associated variables with high accuracy. AKRR identifies all 12 variables with over 88% ratios and identifies 10 out of 12 variables with over 96% ratios when |*w_i_*| = 1. Moreover, the performances are still very impressive when the associations between survival time and covariates are weak. AKRR identifies 10 out of 12 variables with over 95% and 94% ratios when |*w_i_*| = 0.2 and |*w_i_*| = 0.1 respectively. Table 2 gives more details about the average number of variables being selected and the ratios of correctly-detected, over-fitting, and under-fitting. The optimal parameters in the parenthesis are decided by 10-fold cross-validation with the training data only. p* is chosen from the values of 0.6, 0.7, 0.8, 0.9, 1, since our computational experiments show that AKRR seems to converge to the same solution when *p *≥ 0.6 with different initializations for the same data set. λ* is chosen from 0:0.001:1. The average number of selected variables varies from 11.43-12.61 around the true number 12. AKRR identifies exactly the same 12 variables with the ratios of 75%, 54%, and 52% for |*w_i_*| = 1, 0.2, and 0.1 respectively. In all three cases, AKRR chooses the number of variables in the range of 11-13 with over 90% ratio.

**Table 1:** Frequencies of Correctly Identified variables with Different Parameters Out of 100 Simulations

Parameters	|*w_i_*| = 1	|*w_i_*| = 0.2	|*w_i_*| = 0.1
*w*_1_	100	100	99
*w*_11_	100	98	99
*w*_21_	97	99	98
*w*_31_	98	99	99
*w*_41_	98	98	94
*w*_51_	88	77	81
*w*_61_	90	86	77
*w*_71_	98	95	99
*w*_81_	98	98	97
*w*_91_	96	96	95
*w*_101_	99	99	98
*w*_111_	99	100	100

**Table 2:** Model performance with Simulation Data and Different Parameter Values

|*w_i_*|*s *(λ*, p*)	Av. # of Vars	Exactly-match	Overfitting	Underfitting
1 (0.01, 0.6)	12.61	75%	21%	4%
0.2 (0.002, 0.6)	11.52	54%	3%	43%
0.1 (0.001, 0.6)	11.43	52%	2%	46%

For comparison purposes, we also implement the primal version of LASSO for AFT model with Gauss-Seidel method to optimize **w **directly. The computational time for different input dimensions is listed in Table 3. Table 3 shows that AKRR is so computational efficient that it only takes 17.5 seconds for one run to identify variables from 100,000 candidate variables, while LASSO might take days. With 50000 variables, AKRR only needs 7.5 seconds on average to converge, while LASSO fails to converge after 2 hours. When the input dimension is large, AKRR is much more efficient. This is reasonable since the computational time of AKRR is mainly associated with the sample size and dual variables. This method will be fast even with ultra-high dimensional input as long as the sample size is small, which is common in genomic data analysis.

**Table 3:** Computational Time (in Seconds): AKRR vs LASSO

Input Dimensions	AKRR	LASSO
100	0.4801	0.6378
1000	0.5844	6.4577
10000	1.7500	978.23
50000	7.5255	>7200
100000	17.4545	- -

### Diffuse Large B-cell Lymphoma Data

We now consider one diffuse large B-cell lymphoma (DLBCL) data [19] evaluating gene expression profiles associated with the patient's survival. In this study, tumor-biopsy specimens and clinical data were obtained retrospectively from 240 patients with untreated diffuse large-B-cell lymphoma who had no previous history of lymphoma, according to a protocol approved by the National Cancer Institute institutional review board. The median follow-up time was 2.8 years overall (7.3 years for survivors), and 57 percent of patients died during this period. The median age of the patients was 63 years, and 56 percent were men. CDNA microarray data with 7,399 probes were collected. We divide the data into two equal parts with 120 training data and 120 test data. We utilize the two-fold cross validation scheme to choose the optimal *λ *and evaluate our method. We randomly split the data into two roughly equal-sized subsets and build the model with one subset and test it with the other. To avoid the bias arising from a particular partition, the procedure is repeated 100 times, each time we split the data randomly into two folds and do cross validation. The relevance count is utilized to count how many times a gene is selected in the cross validation. Clearly the maximum relevance count for a gene is 200 with the two-fold cross validation and 100 repeating. The optimal λ* is in the range of 0.26-0.3, and the optimal p* is set to 0.7 in all the experiments. The test RRMSE is 0.07 on average, which is better than the average test RRMSE (0.101) with LASSO based primal model. This is reasonable, since AKRR has one additional parameter *p*. Genes associated with survival time are shown in Table 4. We identify 23 probes with over 100 relevant counts. Those 23 probes are corresponding to 21 known genes. All of the selected genes play important roles in apoptotic processes and/or the development and progress of various cancers. 17 out of 21 genes are associated with different lymphoma according to PubMed. For example, BMP6 is the top gene in other category associated with poor outcome and HLA-C gene is from the major histocompatibility class (MHC) II family, both genes were also identified by Rosenwald et al. 2002. Moreover, CD86, CD79a, and CD19 are well known antigens and MHC II signatures associated with favorable survival outcomes. We then perform pathway analysis using DAVID (david.abcc.ncifcrf.gov) and identify 5 lymphoma associated pathways: NOD-like Receptor Signaling Pathway, Pathways in Cancer, Allograft Rejection, Focal Adhesion, and Graft-versus-host Disease. Four out 5 pathways (except for NOD-like Receptor Signaling Pathway) are known to be associated with DLBCL from PubMed.

**Table 4:** Genes Associated with Survival Time for DLBCL Data

Count	GenBank	Symbal	Description
200	X59618	RRM2	ribonucleotide reductase M2 polypeptide
200	X15187	HSP90B1	tumor rejection antigen (gp96) 1
200	M60315	BMP6	bone morphogenetic protein 6
176	U04343	CD86	CD86 antigen (CD28 antigen ligand 2, B7-2 antigen)
181	X07203	MS4A1	membrane-spanning 4-domains, subfamily A, member 2
198	S75217	CD79A	CD79A antigen (immunoglobulin-associated alpha)
200	M28170	SD19	CD19 antigen
138	U45878	BIRC3	baculoviral IAP repeat-containing 3
146	U10485	LRMP	lymphoid-restricted membrane protein
176	U07620	MAPK10	mitogen-activated protein kinase 10
179	LC_30727		
153	M63438	HLA-C	immunoglobulin kappa constant
164	U46767	CCL13	small inducible cytokine subfamily A (Cys-Cys), member 13
142	X14723	CLU	clusterin
200	M27492	IL1R1	interleukin 1 receptor, type I
183	J05070	MMP9	matrix metalloproteinase 9
200	X61118	LMO2	LIM domain only 2 (rhombotin-like 1)
200	M81750	MNDA	myeloid cell nuclear differentiation antigen
115	X57809	IGL@	heat shock 70 kD protein 1A
162	J03746	MGST1	microsomal glutathione S-transferase 1
200	D38535	ITIH4	inter-alpha (globulin) inhibitor H4
200	M21574	PDGFRA	platelet-derived growth factor receptor, alpha polypeptide
187	ESTs	ESTs	

### Follicular Lymphoma (FL) Data

Follicular lymphoma is a common type of Non-Hodgkin Lymphoma (NHL). It is a slow growing lymphoma that arises from B-cells, a type of white blood cell. It is also called an "indolent" or "low-grade" lymphoma for its slow nature, both in terms of its behavior and how it looks under the microscope. A study was conducted to predict the survival probability of patients with gene expression profiles of tumors at diagnosis [20]. Fresh-frozen tumor biopsy specimens and clinical data were obtained from 191 untreated patients who had received a diagnosis of follicular lymphoma between 1974 and 2001. The median age of patients at diagnosis was 51 years (range 23 - 81) and the median follow up time was 6.6 years (range less than 1.0 - 28.2). The median follow up time among patients alive was 8.1 years. Four records with missing survival information were excluded from the analysis. Affymetrix U133A abd U133B microarray genechips were used to measure gene expression levels from RNA samples. A log 2 transformation was applied to the Affymetrix measurement. Detailed experimental protocol can be found from the original paper. There are total of 42928 probes. It is time consuming to directly apply standard LASSO methods to this problem without an initial reduction of dimensions. Our method takes less than 10 seconds for one run. Similar two-fold cross validation scheme with 100 random partitions is utilized to this data. The optimal λ* is in the range of 0.1 - 0.12 with the optimal p* = 0.6. The test RRMSE is 0.09. The final results are shown in Table 5.

**Table 5:** Genes Associated with Survival Time for FL Data

count	ProbeID	Symbal	Description
200	231760_at	C20orf51	chromosome 20 open reading frame 51
200	232932_at		
200	235856_at	C4A	complement component 4A (Rodgers blood group)
187	224280_s_a	LOC56181	family with sequence similarity 54, member B
200	201425_at	ALDH2	aldehyde dehydrogenase 2 family (mitochondrial)
180	214694_at	M-RIP	Myosin phosphatase Rho-interacting protein
200	214713_at	YLPM1	YLP motif containing 1
200	218477_at	TMEM14A	transmembrane protein 14A
200	220669_at	HSHIN1	HIV-1 induced protein HIN-1
195	203970_s_a	PEX3	peroxisomal biogenesis factor 3
200	208470_s_a	HPR	haptoglobin-related protein; haptoglobin
175	210920_x_a		
200	215444_s_a	TRIM31	tripartite motif-containing 31

Thirteen probes with over 100 relevance counts are identified. Those 13 probes are corresponding to 11 known genes associated with lymphoma and related diseases. For instance, gene C4A localizes to the major histocompatibility complex (MHC) class III region on chromosome 6. It plays a central role in the activation of the classical pathway of the complement system. C4A anaphylatoxin is a mediator of local inflammatory process. It induces the contraction of smooth muscle, increases vascular permeability, and causes histamine release from mast cells and basophilic leukocytes. C4A is on the pathway of Systemic Lupus Erythematosus (SLE). Patients with SLE can increase the risk of certain cancers, including non-Hodgkin's lymphoma. We find that C4A is negatively associated with survival time according the estimated coefficient of C4A. ALDH2 is another well studied gene which is significantly associated with acetaldehyde-induced micronuclei and alcohol-induced facial flushing. Defects in ALDH2 are a cause of acute alcohol sensitivity and alcohol induced cancers. There are accumulating evidences that ALDH2-deficient individuals are at much higher risk of esophageal cancer and malignant lymphoma. Our study indicates that the up-regulated ALDH2 is positively associated with patient survival outcomes. Six other genes are also associated with different cancers including follicular lymphoma.

## Conclusions

We proposed kernel based methods for nonlinear AFT model and variable selection for ultra-high dimensional data. Our evaluations with simulation and real data illustrate that the proposed methods can effectively reduce the dimension of the covariates with sound prediction accuracy. In many studies, both clinical and genomic data are available. Due to the ultra-high dimension in genomic data, directly applying LASSO based methods to genomic data is usually not feasible. Our proposed method provides an efficient solution for it. Kernel based nonparametric methods have been well studied in statistical learning, but there are not many studies for survival analysis. In this paper, we provide a basis for further explorations in this field.

## Methods

To formulate the model, consider a set of n independent observations ${\left\{T_{i},\;\delta_{i},\;x_{i}\right\}}_{i=1}^{n}$, where *δ_i _*is the censoring indicator, *T_i _*is the survival time (event time) if *δ_i _*= 1 or censoring time if *δ_i _*= 0, and **x***_i _*= (*x*_*i*1_, *x*_*i*2_, ..., *x_im_*)*^t ^*is the m-dimensional input vector of the *i*th sample. Letting **w **= (*w*_1_, *w*_2_, ..., *w_m_*)*^t ^*be a vector of regression coefficients and *ϕ*(**x***_i_*) is the nonlinear transform of **x***_i _*in feature space, the AFT model is defined as

(1) $$\begin{array}{cc} M\left(x_{i}\right)=w^{t}\varphi \left(x_{i}\right), & i=1,\;...,\;n, \end{array}$$

where *M*(**x***_i_*) > log *T_i _*if *δ_i _*= 0 and *M*(**x***_i_*) = log *T_i _*if *δ_i _*= 1. Because there are both equality and inequality constraints in the model, new methods need to be developed.

### Kernel Ridge Regression (KRR)

The kernel ridge regression for right censored survival data is as follows:

(2) $$\begin{array}{l} \;\min\frac{1}{2n}\sum_{i=1}^{n}\xi_{i}^{2}+\frac{\lambda }{2}w^{t}w\\ \text{s}.\text{t}.\;|w^{t}\varphi \left(x_{i}\right)-\log T_{i}|<\xi_{i},\\ \;\;\text{if}\;\delta_{i}=1;\\ \;\;w^{t}\varphi \left(x_{i}\right)>\log T_{i}-\xi_{i},\\ \;\;\text{if}\;\delta_{i}=0;\\ \;\;\xi_{i}\geq 0,\;\forall \;\;1\leq i\leq n. \end{array}$$

When ties in the event times are presented, variables associated with each tied time appear in the constraints independently. We can define an index function *I*(*δ_i_*) = 1 if *δ_i _*= 1, and for censored data with *δ_i _*= 0, *I*(*δ_i_*) is defined as *I*(*δ_i_*) = 1 if log *T_i _*≥ **w***^t^ϕ*(**x***_i_*) and 0 otherwise. Then equation (2) is equivalent to the following quadratic function:

(3) $$\begin{array}{l} J\left(w\right)=\\ \;\frac{1}{2n}\sum_{i=1}^{n}I\left(\delta_{i}\right){\left\{w^{t}\varphi \left(x_{i}\right)-\log T_{i}\right\}}^{2}\\ \;+\frac{\lambda }{2}w^{t}w, \end{array}$$

where *λ *≥ 0. If we set the gradient of *J*(**w**) with respect to **w **to zero, then the solution for **w **is a linear combination of the vectors *ϕ*(**x***_i_*):

(4) $$\begin{array}{l} w=\\ -\frac{1}{n\lambda }\sum_{i=1}^{n}I\left(\delta_{i}\right)\left\{w^{t}\varphi \left(x_{i}\right)-\log T_{i}\right\}\varphi \left(x_{i}\right)\\ =\sum_{i=1}^{n}a_{i}\varphi \left(x_{i}\right)=\Phi^{t}a, \end{array}$$

where Φ is the design matrix, whose *i^th ^*row is given by *ϕ*(**x***_i_*)*^t^*, and **a **= (*a*_1_, *a*_2_, ..., *a_n_*)*^t ^*are the dual variables, defined by

(5) $$a_{i}=-\frac{I\left(\delta_{i}\right)}{n\lambda }\left\{w^{t}\varphi \left(x_{i}\right)-\log T_{i}\right\}$$

Substituting **w **= Φ*^t^***a **into *a_i_*, we obtain

(6) $$\begin{array}{c} a_{i}=-\frac{I\left(\delta_{i}\right)}{n\lambda }\left\{\varphi^{t}\left(x_{i}\right)\Phi^{t}a-\log T_{i}\right\}\\ =-\frac{I\left(\delta_{i}\right)}{n\lambda }\left\{K\left(x_{i},\;.\right)a-\log T_{i}\right\}, \end{array}$$

where *K *= (*K*(**x***_i_*, **x***_j_*))*_n × n _*= (*ϕ*(**x***_i_*)*^t^ϕ*(**x***_j_*))*_n × n _*is a kernel matrix which can be defined explicitly and *K*(**x***_i_*,.) = *ϕ*(**x***_i_*)*^t^*Φ*^t ^*is the *i*-th row of the kernel matrix. Popular kernels include:

• Linear kernel:

$$K\left(x_{i},\;x_{j}\right)=x_{i}^{t}x_{j},$$

• Radial basis function (Gaussian) kernel:

$$K\left(x_{i},\;x_{j}\right)=\exp\left(-\frac{|x_{i}-x_{j}{|}^{2}}{\sigma^{2}}\right),$$

• Polynomial kernel:

$$K\left(x_{i},\;x_{j}\right)={\left(x_{i}^{t}x_{j}+p_{2}\right)}^{p_{1}},$$

• Sigmoid kernel:

$$K\left(x_{i},\;x_{j}\right)=\tanh\left(\beta x_{i}^{t}x_{j}\right).$$

Our kernel ridge regression algorithm based on the dual equation (DKRR) (6) is as follows:

#### The Dual Kernel Ridge Regression (DKRR) Algorithm

Given *λ*, training data ${\left\{x_{i},\;\log T_{i},\;\delta_{i}\right\}}_{i=1}^{n}$, test data ${\left\{x_{k},\;\log T_{k},\;\delta_{k}\right\}}_{k=1}^{n_{k}}$, and a small *ε*.

Calculate the kernel matrices $K={\left[K\left(x_{i},\;x_{j}\right)\right]}_{n\times n}$ and $K_{te}={\left[K\left(x_{k},\;x_{i}\right)\right]}_{n_{k}\times n}$.

Center the kernels and the survival times: $K=\left(I_{n}-\frac{1}{n}1_{n}1_{n}^{t}\right)K\left(I_{n}-\frac{1}{n}1_{n}1_{n}^{t}\right)$ and $K_{te}=\left(K_{te}-\frac{1}{n}1_{n_{k}}1_{n}^{t}K\right)\left(I_{n}-\frac{1}{n}1_{n}1_{n}^{t}\right)$, where 1*_n_*: a vector with n 1's. and *I_n_*: an identity matrix, and $\log T_{k}=\log T_{k}-\frac{\sum_{k=1}^{n_{k}}\log T_{k}}{n_{k}}$. Let **a**^0 ^= [0, ..., 0, 0]*^t^*, and *j *= 0

WHILE 1,

• FOR *i *= 1 to *n*,

            $I\left(\delta_{i}\right)=\{\begin{array}{ll} 1 & \text{if }\delta_{i}>0,\\ 1 & \text{if }\delta_{i}=0,\\ & \&\;K\left(x_{i},.\right)a^{j}\leq \log T_{i},\\ 0 & \text{otherwise}. \end{array}$

            $a_{i}^{j+1}=-\frac{I\left(\delta_{i}\right)}{n\lambda }\left\{K\left(x_{i},\;.\right)a^{j}-\log T_{i}\right\}$

            $a^{j+1}={\left[a_{1}^{j+1},\;...,\;a_{i}^{j+1},\;a_{i+1}^{j}...,\;a_{n}^{j}\right]}^{t}$

         END

• *j *= *j *+ 1

• IF |**a***^j+1 ^*- **a***^j^| *<*ε*, BREAK.

• **a***^j ^*= **a**^*j*+1^

END

This dual kernel ridge regression (DKRR)algorithm designed for a quadratic error function with linear constraints is a convex function with convex constraints. Theoretically this algorithm will always converge and global optimal solution is guaranteed irrelevant to initial value **a**^0^. In our computational experiments, the differences of the estimated parameters with different initial values are very small (less than 0.01 with the infinity norm).

### Adaptive Kernel Ridge Regression

When the number of variables is greater than the sample size *n*, regularization is needed to obtain a stable estimator **w**. We propose a $L_{p}=\sum_{i=1}^{n}\left|w_{i}{|}^{p}(p<1\right)$ penalty for variable selection and estimation simultaneously. Unlike LASSO, *L_p _*penalty and its different approximation schemes (i.e., adaptive LASSO) possess the oracle property [21,22]. Here the oracle property of a method means that it can correctly identify the nonzero coefficients with probability converging to one and that the estimators of nonzero coefficients are asymptotically normal with the same means and covariances as what they would have the zero coefficients be known in advance. We therefore propose the following penalized AFT model:

(7) $$\begin{array}{l} J\left(w\right)=\\ \;\frac{1}{2n}\sum_{i=1}^{n}I\left(\delta_{i}\right){\left\{w^{t}\varphi \left(x_{i}\right)-\log T_{i}\right\}}^{2}\\ \;+\frac{\lambda }{2}\sum_{i=1}^{m}|w_{i}{|}^{p}\\ \;=\frac{1}{2n}\sum_{i=1}^{n}I\left(\delta_{i}\right){\left\{w^{t}\varphi \left(x_{i}\right)-\log T_{i}\right\}}^{2}\\ \;+\frac{\lambda }{2}\sum_{i=1}^{m}\frac{|w_{i}{|}^{2}}{|w_{i}{|}^{2-p}}, \end{array}$$

where *λ *≥ 0. With *n *≪ *m*, linear kernel is more appropriate, since model with linear kernel has less over-fitting. We will take *ϕ*(**x***_i_*) = **x***_i_*, introduce an auxiliary (latent) variable vector **u **= [*u*_1_, *u*_2_, ..., *u_m_*]*^t^*, and develop an adaptive procedure based on equation (7). Equation (7) can be rewritten as:

(8) $$\begin{array}{l} J\left(w,\;u\right)=\\ \;\frac{1}{2n}\sum_{i=1}^{n}I\left(\delta_{i}\right){\left\{w^{t}x_{i}-\log T_{i}\right\}}^{2}\\ \;+\frac{\lambda }{2}\sum_{i=1}^{m}\frac{|w_{i}{|}^{2}}{|u_{i}{|}^{2-p}}, \end{array}$$

(9) and, u=w.

With equation (8) and (9), we will find the first order derivative for **w **with a fixed **u **and then update **u **= **w**. After taking the first order derivative, we have the following equation:

(10) $$\begin{array}{l} w=\\ \;-\frac{1}{n\lambda }\sum_{i=1}^{n}I\left(\delta_{i}\right)\left\{w^{t}x_{i}-\log T_{i}\right\}\left(x_{i}⊙|u{|}^{2-p}\right)\\ \;=\sum_{i=1}^{n}a_{i}\left(x_{i}⊙|u{|}^{2-p}\right)=X_{u}^{t}a,\;\; \end{array}$$

where ⊙ represents the componentwise product of two vectors and

$$X_{u}=\;\left(\begin{array}{cc} x_{1}^{t}\;\;\;\;⊙ & {\left(|u{|}^{2-p}\right)}^{t}\\ & \vdots \\ x_{n}^{t}\;\;\;\;\;⊙ & {\left(|u{|}^{2-p}\right)}^{t} \end{array}\right),$$

and

(11) $$a_{i}=-\frac{I\left(\delta_{i}\right)}{n\lambda }\left\{w^{t}x_{i}-\log T_{i}\right\}.$$

We substitute $w=X_{u}^{t}a$ and define a new kernel function $K_{u}=XX_{u}^{t}$. Then we have $K_{u}\left(x_{i},.\right)=x_{i}^{t}X_{u}^{t}$, which is the ith row of *K*_**u**_. So,

(12) $$\begin{array}{c} a_{i}=\;-\frac{I\left(\delta_{i}\right)}{n\lambda }\left\{x_{i}^{t}X_{u}^{t}a-\log T_{i}\right\}\\ =\;-\frac{I\left(\delta_{i}\right)}{n\lambda }\left\{K_{u}\left(x_{i},.\right)a-\log T_{i}\right\}. \end{array}$$

The adaptive kernel ridge regression algorithm based on dual variables **a **with equation (10) and (12) is as follows:

#### Adaptive Kernel Ridge Regression (AKRR) Algorithm

Given a *λ*, *p *∈ (0.1], training data ${\left\{x_{i},\;\log T_{i},\;\delta_{i}\right\}}_{i=1}^{n}$, and a small *ε *and *η*.

Initializing **w **= **u **= rand(*m*, 1), and **a **= [0, ..., 0]*^t^*

Setting **u**(*u_i _*== 0) = 10*e *- 5 and *j *= 1.

While |**w **- **u**| >*ε*

• **u **= **w**,

• $K_{u}=XX_{u}^{t}$

• FOR *i *= 1 to *n*,

            $I(\delta_{i})=\{\begin{array}{ll} 1 & \text{if }\delta_{i}>0\\ 1 & \text{if }\delta_{i}=0,\\ & \&\;K\left(x_{i},.\right)a^{j}\leq \log T_{i},\\ 0 & \text{otherwise}. \end{array}$

            $a_{i}^{j+1}=-\frac{I\left(\delta_{i}\right)}{n\lambda }\left\{K_{u}\left(x_{i},\;.\right)a^{j}-\log T_{i}\right\}$

            $a^{j+1}={\left[a_{1}^{j+1},\;...,\;a_{i}^{j+1},\;a_{i+1}^{j}...,\;a_{n}^{j}\right]}^{t}$

         END

• *j *= *j *+ 1

• $w=X_{u}^{t}a$.

END

**w**(**w **<*η*) = 0

Unlike other LASSO based methods which seek to find optimal **w **directly, AKRR algorithm updates the m-dimensional **w **through updating a much smaller n-dimensional dual variable **a**. This method is computationally highly efficient when *n *≪ *m*, which is common in genomic data. Although the proposed method is based on the dual problem, the primal variable **w **is explicitly updated in the computation. Theoretically AKRR algorithm will always converge to global optimal solution when *p *= 1 irrelevant to initial values of **w**, **u**, and **a**, as the error function is convex under *L*_1 _penalty, but only local optimal solution is guaranteed when *p *< 1. However, in our computational experiments with simulation and real data, even though we may have different optimal solutions with different initializations only when *p *≤ 0.5, most selected features are still the same in different runs. AKRR always reach the same optimal solution in all of our experiments when *p *≥ 0.6. One possible explanation is that the error function may still be near convex or convex almost everywhere when *p *is large. Therefore it may be possible that we enjoy both the oracle property with less bias and the global optimal solution with larger *p *(0.6 ≤ *p *< 1). Theoretical study for the near convex error function, however, is out of the scope of this paper. To prevent the results stick to a local optimal solution when *p *≤ 0.5, we run AKRR 30 times and the best solution is chosen from the run with smallest test error. Even though AKRR does choose different variables with different *p*s, a small subset (≥ 5) of most important genes are always selected in our experiments. The model performance can be evaluated with cross-validation and the relative root mean squared error $\left(\text{RRMSE}=\sqrt{\frac{\sum_{i}{\left((y_{i}-{\hat{y}}_{i})/y_{i}\right)}^{2}}{n}}\right)$ of the test data. There are two parameters *p *and *λ *for the adaptive kernel ridge regression (AKRR) algorithm. One efficient way is to set *p *= 0.1, 0.2, ..., 1 alternatively, and then search for the best *λ *through cross-validation. The range of can be determined by the path of the optimal solution. *λ*_min _= 0 and *λ*_max _is set to be the smallest value with all zero estimated parameters by multiple trials. We search the optimal *λ *from *λ *∈ (0, 1] in this paper. Usually we have a larger *λ*_max _for *p *= 1, and smaller *λ*_max _when *p *is smaller.

## Authors' contributions

ZL conceptualized and designed method, developed the software, and wrote the manuscript. FJ and RG analyzed and interpreted the data on its biological contents. DC and MT helped in method design and manuscript writing and revised the manuscript critically. All authors read and approved the final manuscript.
